# Editorial: Biotechnologies to recover critical metals

**DOI:** 10.3389/fbioe.2025.1747699

**Published:** 2025-12-08

**Authors:** Denys Kristalia Villa Gomez, Eric D. van Hullebusch, Aurora M. Pat Espadas, Iván Nancucheo

**Affiliations:** 1 School of Civil Engineering, The University of Queensland, Brisbane, QLD, Australia; 2 Australian Institute for Bioengineering and Nanotechnology, The University of Queensland, Brisbane, QLD, Australia; 3 Institut de Physique du Globe de Paris, CNRS, Université Paris Cité, Paris, France; 4 Departamento de Ingeniería Química y Metalurgia, Universidad de Sonora, Hermosillo, Mexico; 5 Universidad San Sebastián, Concepción, Chile

**Keywords:** biotechnolgy, circular economy, critical metals recovery, bioleaching, biosorption, rare earth

## Introduction

Critical metals, ranging from rare earth elements (REEs) to cobalt, nickel, vanadium, and gallium, are indispensable to modern infrastructure and the global energy transition. Their low natural abundance and geographic concentration create significant supply risks, while conventional extraction methods are energy-intensive and environmentally damaging. At the same time, anthropogenic deposits such as industrial residues, mine tailings, spent catalysts, and waste electrical and electronic equipment contain substantial quantities of these valuable elements. Recovering metals from these secondary resources offers an opportunity to reduce waste, enhance resource circularity, and strengthen strategic metal security.

However, secondary recovery is not without challenges: extraction processes must avoid secondary pollution, minimize chemical inputs, and maintain low energy consumption. Within this context, biotechnology has emerged as a powerful and versatile tool capable of addressing these limitations. Bioleaching, biosorption, biomineralization, enzymatic processes, and protein-based metal binding collectively provide selective, low-impact, and adaptable routes for metal recovery from complex matrices.

The compiled Research Topic illustrates the breadth and maturity of biotechnological innovations applied to the recovery of critical metals, focusing on targeted valorisation of key industrial and urban waste streams. These studies present innovative bio-strategies ranging from bioleaching to biosorption, demonstrating significant advances in sustainable, efficient recovery methods that contribute to the circular economy and the sustainable management of critical raw materials.

Industrial residues such as bauxite residue, iron ore concentrates, coal fly ash, and electronic waste, once considered environmental liabilities, are now recognized as valuable reservoirs of critical metals. The comprehensive review on bauxite residue valorisation highlights microbial bioleaching approaches to extract rare earth elements, gallium, vanadium, and titanium by leveraging optimal microorganisms and metabolic pathway optimization to enhance recovery efficiency, reinforcing circular economy principles (Soto et al.). Complementing this, Bobadilla-Fazzini and Poblete-Castro demonstrate the use of *Acidithiobacillus thiooxidans* for sulfur removal from iron ore concentrates. Operating stirred-tank and packed-column bioreactors at 30 °C, they achieved up to 80% desulfurization, offering a clean alternative to conventional chemical processes and highlighting the role of bioreactor configuration in optimizing microbial performance.

Advancing molecular and protein-based recovery strategies, Techert et al. and Hussain et al. demonstrate the remarkable selectivity and reusability of lanmodulin-inspired peptides and elastin-like polypeptides (RELPs) in recovering REE. Techert et al. employ phage surface display and next-generation sequencing to identify europium-binding peptides, with the GC9 sequence showing exceptional selectivity. Such peptide-based ligands could replace traditional chemical extractants with recyclable, biodegradable alternatives. On the other hand, Hussain et al. develop thermo-responsive elastin-like polypeptides (RELPs) able to extract REEs from coal fly ash with high selectivity and reusability, achieving a 100,000-fold increase in purity while maintaining 95% binding capacity across multiple cycles.

Finally, waste-derived biosorbents employing abundant and low-cost biomasses reveal promising selective recovery of multiple metals from complex electronic waste leachates. Sieber et al. repurpose spent brewer’s yeast as a low-cost biosorbent to recover metals from polymetallic waste streams. Their design-of-experiments approach reveals strong pH-dependent selectivity, achieving greater than 90% zinc and greater than 50% copper recovery while demonstrating biomass reusability across multiple cycles.

Collectively, these studies represent a continuum of biological innovation, from microbial desulfurization and bioleaching to advanced peptide engineering and biomass-based sorption, illustrating how biotechnology can intervene at every stage of the metal life cycle. They also highlight the dynamism of the field and the growing integration of omics, artificial intelligence, combinatorial biotechnology, and adaptive evolution to design next-generation bioprocesses.

Realising the full potential of biological metal recovery will require interdisciplinary collaboration, methodological innovation, and pilot-scale validation. Hybrid systems that combine biological selectivity with industrial throughput, along with deeper exploration of alkaline residues and underutilized waste streams, will be essential to transforming waste management into resource generation. Continued exchange across microbiology, bioprocess engineering, materials science, and industrial ecology will help overcome remaining challenges and accelerate the deployment of environmentally benign, scalable recovery technologies.

## Conclusion

The contributions assembled in *“Biotechnologies to Recover Critical Metals”* reflect a transformative vision: shifting from extractive, energy-intensive mining to bio-inspired processes that regenerate value from waste, promote circularity, and support a sustainable and resilient future for critical metals. By advancing microbial, molecular, and biomass-based strategies, this Research Topic underscores the central role biotechnology will play in securing the materials essential for the green transition and the technological evolution of modern society.

